# Long Non-Coding RNA CTD-2245E15.3 Drives Proliferation and Migration in Gastrointestinal Stromal Tumors

**DOI:** 10.3390/biomedicines14030514

**Published:** 2026-02-26

**Authors:** Xiangfei Sun, Yinwen Sun, Ping Shu, Tuo Yi, Kuntang Shen, Weixin Niu, Xinqiang Hong

**Affiliations:** Department of General Surgery, Zhongshan Hospital, Fudan University School of Medicine, Shanghai 200032, China; sun.xiangfei@zs-hospital.sh.cn (X.S.); 23211210061@m.fudan.edu.cn (Y.S.); shu.ping@zs-hospital.sh.cn (P.S.); yi.tuo@zs-hospital.sh.cn (T.Y.); shen.kuntang@zs-hospital.sh.cn (K.S.)

**Keywords:** long non-coding RNA, gastrointestinal stromal tumor, high content screening, proliferation, migration

## Abstract

**Background:** Long non-coding RNAs (lncRNAs) participate in a wide range of physiological processes, and their dysregulation is prevalent in human cancers, indicating critical roles in tumorigenesis. In intermediate- to high-risk gastrointestinal stromal tumors (GISTs), resistance to tyrosine kinase inhibitors (TKIs) remains a major therapeutic challenge. Therefore, identifying lncRNAs as potential novel therapeutic targets is of considerable interest. **Methods:** Three paired samples of intermediate- to high-risk GIST tissues and adjacent normal tissues were subjected to transcriptome sequencing. High-content screening (HCS) was subsequently performed to identify candidate lncRNAs with significant effects on GIST cell proliferation. Loss-of-function experiments were conducted, and cell proliferation, migration, and apoptosis were evaluated using the Cell Counting Kit-8 (CCK-8) assay, colony formation assay, Transwell migration assay, and flow cytometry, respectively. In addition, in situ hybridization (ISH) was performed on 507 primary GIST tissue specimens to examine the association between CTD-2245E15.3 expression and clinicopathological features, including progression-free survival (PFS) and overall survival (OS). **Results:** Transcriptome sequencing revealed 2924 upregulated and 2629 downregulated lncRNAs in GIST tissues compared with adjacent normal tissues. Based on HCS results, CTD-2245E15.3 was selected for further functional analyses. CCK-8 assays demonstrated that knockdown of CTD-2245E15.3 significantly inhibited proliferation of GIST cells. Consistently, colony formation and migratory capacity were markedly reduced in the shCTD-2245E15.3 group compared with controls. Furthermore, flow cytometric analysis showed a significant increase in apoptosis following CTD-2245E15.3 silencing. ISH analysis revealed that high CTD-2245E15.3 expression correlated with adverse clinicopathological features and poorer PFS and OS. **Conclusions:** Our study demonstrates that CTD-2245E15.3 promotes proliferation and migration of GIST cells and is associated with poor prognosis, highlighting its potential as a therapeutic target and diagnostic biomarker.

## 1. Introduction

Gastrointestinal stromal tumors (GISTs) are the most common mesenchymal tumor of the gastrointestinal tract [1], mostly occurring in the stomach and small intestine, and less commonly in the esophagus, colorectum and outside the gastrointestinal tract. The GIST annual incidence is approximately 1–2/100,000, with no significant sex or racial differences [2]. Depending on the size and location of the tumor, common clinical symptoms include abdominal pain, bloating, dysphagia, bleeding, and obstruction in the digestive tract. GISTs are highly heterogeneous, with the incidence of micro GISTs in the elderly population being as high as 30% with little or no malignant potential, while large and high mitotic count GISTs are at high risk of recurrence and prone to peritoneal metastases, which are often fatal [3].

At the beginning of this century, the median survival time of patients with advanced GIST was approximately one year [4]. The subsequent introduction of imatinib, a tyrosine kinase inhibitor (TKI), significantly improved outcomes for intermediate- and high-risk GIST patients [5,6]; however, many patients develop resistance within 1–3 years [2]. The second- and third-line multitargeted TKIs sunitinib [7] and regorafenib [8] only provide limited improvement in prognosis, with a median progression-free survival (PFS) that is only 4–5 months longer compared to placebo. Therefore, there is an urgent requirement to further explore the GIST’s pathogenesis and find potential new therapeutic targets.

Long non-coding RNA (lncRNA) is defined as non-coding RNA greater than 200 nucleotides in length. LncRNAs can influence gene expression at various epigenetic, transcriptional and post-transcriptional levels and play an oncogenic and/or cancer-inhibiting role in neoplastic diseases [9,10]. Moreover, lncRNAs also play an important role in tumor metastasis, and are involved in multiple processes including local invasion, epithelial-mesenchymal transition (EMT), metastatic colonization, regulation of organ-specific tropism, and the metastatic microenvironment [11]. In addition, most lncRNAs are expressed with tissue and cell-type specificity [12] and thus have theoretical potential to become efficient targets for tumor therapy. Moreover, their high tissue specificity and ability to regulate specific facets of the cellular networks may make lncRNAs superior to proteins in terms of their potential targeting-related adverse effects. Features such as lack of translation, rapid turnover, and low basal expression levels allow lncRNAs to exert effects at low doses [13]. These features have made lncRNAs one of the foci of oncology research.

To better elucidate the potential roles of lncRNAs in the progression of intermediate- to high-risk GISTs and to identify novel therapeutic targets or biomarkers, we conducted a comprehensive lncRNA expression profiling study. Specifically, three pairs of imatinib-untreated intermediate- to high-risk GISTs and matched peritumoral tissues were sequenced for lncRNAs and subjected to differential analysis and GO/KEGG enrichment analysis to explore the possible involvement of differentially expressed genes in biochemical, metabolic, and signal transduction pathways. Subsequently, high-content screening (HCS) was employed to identify lncRNAs that significantly promote cell proliferation, leading to the selection of CTD-2245E15.3 for functional validation as a potential therapeutic target or biomarker.

## 2. Materials and Methods

### 2.1. Clinical Specimens

All patient samples were collected from Zhongshan Hospital, Fudan University, whose medical ethics committee has reviewed and approved this study. We collected three pairs of fresh frozen specimens of primary GISTs and paratumor normal tissues with different locations for lncRNA sequencing. The detailed clinicopathological characteristics of the three pairs of sequenced samples are listed in Table 1. The pathological diagnosis for each case was confirmed by at least two senior pathologists. A total of 507 primary GIST tissue specimens were collected for RNA in situ hybridization (ISH) analysis of CTD-2245E15.3 expression. Patients were stratified into high- and low-expression groups according to the median ISH score. Clinicopathological characteristics and survival outcomes were compared between the two groups. PFS and overall survival (OS) were obtained through regular outpatient visits and telephone follow-up. PFS was calculated from the date of initial tumor resection to the occurrence of documented disease progression or the date of last follow-up. OS was calculated from the date of surgery to death from any cause or the last known follow-up.

### 2.2. Library Construction and Sequencing Process

We extracted RNAs from the tissues using Trizol reagent (Invitrogen, Carlsbad, CA, USA) and applied agarose gel electrophoresis to analyze the RNA integrity and possible presence of DNA contamination [14]. The RNA concentration and purity were then checked initially by Nanodrop 2000 (Thermo Fisher, Wilmington, DE, USA) and precisely by the Agilent 2100 bioanalyzer (Agilent, Santa Clara, CA, USA) for RNA integrity. Then, ribosome RNAs were removed from the total RNA using the rRNA Depletion Kit (NEBNext, Ipswich, MA, USA) according to the manufacturer’s instructions, and strand-specific libraries were constructed. After the libraries were qualified, Illumina PE150 sequencing (Illumina, Inc., San Diego, CA, USA) was performed after pooling according to the effective concentration of the libraries and data output requirements.

### 2.3. Sequencing and Screening of Hub lncRNAs

**Figure 1:**
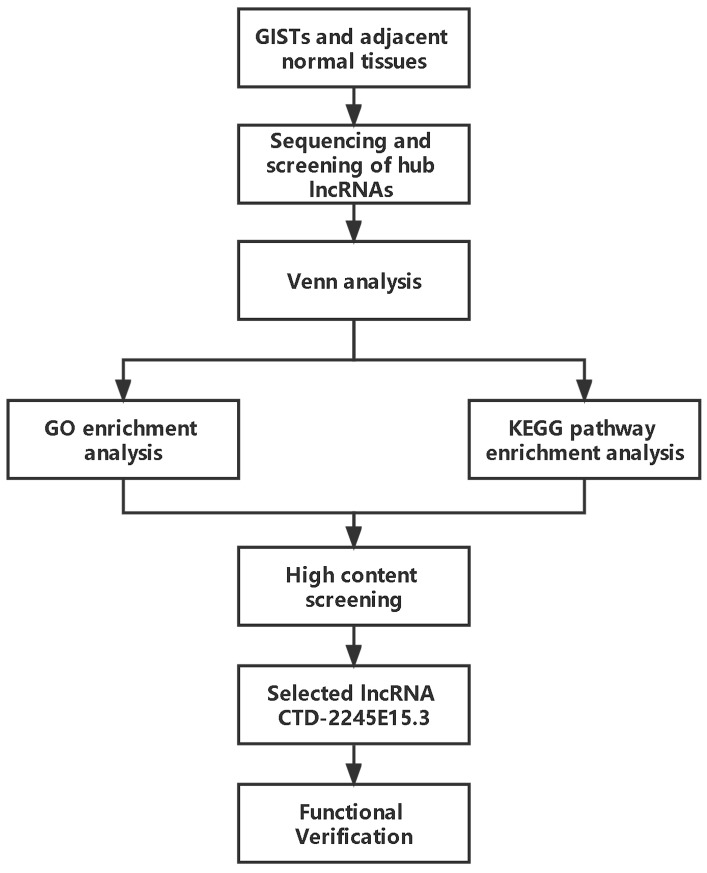
Workflow for identifying lncRNA networks involved in gastrointestinal stromal tumors. GISTs, gastrointestinal stromal tumors; lncRNA, long non-coding RNA.

The lncRNA screening workflow is illustrated in Figure 1. Following transcriptome sequencing, differentially expressed lncRNAs between tumor and adjacent normal tissues were identified using the criteria |log2FC| ≥ 1 and adjusted *p* value (Padj) < 0.05, with *p* values corrected by the Benjamini–Hochberg method to control the false discovery rate (FDR). Venn analysis was subsequently performed to identify lncRNAs that were consistently upregulated or downregulated across all three paired samples.

### 2.4. Cluster Analysis

Cluster analysis is another way to display differentially expressed genes, which brings together those with similar expression patterns. These genes may have common functions or participate in common metabolic and signal pathways. The values of log10 (FPKM + 1) were transformed and analyzed by cluster analysis.

### 2.5. Gene Ontology (GO) and KEGG Pathway Enrichment Analysis

The GO database can describe gene and protein functions in detail by tree stratification and can clarify the hierarchical relationship between gene functions, which are divided into molecular functions (MFs), biological processes (BPs), and cellular components (CCs). GO enrichment analysis is based on the GO database. The differential genes are analyzed by Fisher’s exact test, and the target functional genes are analyzed statistically. KEGG is a database for systematic analysis of gene function and genome information, and signal pathway analysis is based on this database. The pathways involved in the target genes were statistically analyzed by Fisher’s exact test, and the signal pathways with significant differences were screened out according to Padj < 0.05.

### 2.6. High-Content Screening (HCS)

A high-content screening (HCS) approach was applied as a data-driven strategy to prioritize lncRNAs with potential functional relevance in GIST cells. For each candidate gene, three RNA interference targets were designed, and the corresponding plasmids were mixed in equal proportions for lentiviral packaging. GIST-T1 cells were seeded into 96-well plates at a density of 2 × 10^3^ cells per well and infected with appropriate amounts of lentivirus. The list of lentiviral constructs used in the screening is shown in Table 2. Cell proliferation was monitored once daily for seven consecutive days using the Celigo imaging cytometer (Nexcelom, Lawrence, MA, USA). Cells expressing green fluorescence were automatically identified, photographed, and quantified. Cell proliferation fold changes were calculated for each group at each time point, and growth curves were generated to evaluate the effects of lncRNA knockdown on cell proliferation. Candidate lncRNAs were initially prioritized based on a predefined proliferation fold-change threshold and the consistency of their growth-inhibitory effects across time points. CTD-2245E15.3 was selected for subsequent functional validation based on its robust and reproducible anti-proliferative phenotype in the HCS assay, together with efficient knockdown performance.

### 2.7. Cell Culture and Lentiviral Transduction

The GIST-T1 cell line (harboring a KIT exon 11: V560_Y578del mutation, RRID: CVCL_7044) was purchased from Cosmo Bio Co., Ltd. (Tokyo, Japan). The GIST-882 cell line (bearing a KIT exon 13 K642E mutation) was kindly provided by Dr. Fletcher (Harvard Medical School). GIST-T1 cells were cultured in Iscove’s Modified Dulbecco’s Medium (IMDM, Corning, New York, NY, USA), whereas GIST-882 cells were maintained in RPMI-1640 medium (Corning, USA). Both media were supplemented with 10% fetal bovine serum (FBS, Ausbian) in a 37 °C incubator containing 5% CO_2_. Short hairpin RNAs (shRNAs) targeting lncRNAs and control lncRNAs were synthesized by GeneChem (Shanghai, China). shCTD-2245E15.3 (5′-GCATTCTTAGGAAATGCCTCC-3′) was inserted into the GV112 lentiviral vector and a non-silencing shRNA was designed as a negative control (shCtrl group). The cells were infected with lenti-shlncRNA or lenti-shCtrl as recommended by the manufacturer. Briefly, GIST-T1 cells were incubated with viral supernatants for 72 h, then the infected cells were selected with puromycin (2 µg/mL) for 2 days before the experiments. The knockdown efficacy was determined using quantitative polymerase chain reaction (qPCR).

### 2.8. Cell Proliferation

CCK-8 reagent (Dojindo, Kumamoto, Japan) was used to measure GIST-T1 cell proliferation. The GIST-T1 cells were seeded in 96-well plates with 2 × 10^3^ cells/well. Two hours before detection, 10 µL of CCK reagent was added to each well, and then the absorbance value at 450 nm was measured. Each experiment had three replicates, was repeated three times, and was measured continuously for 5 days.

### 2.9. Cell Apoptosis Analysis

The cells were seeded in 6-well plates at a density of 1 × 10^5^ cells per well and cultured for 2 days. Cell apoptosis was detected with an apoptosis assay kit (eBioscience, San Diego, CA, USA) according to the instructions. Briefly, cells in 6-well plates were harvested with 0.25% trypsin-EDTA, washed twice with cold phosphate-buffered saline (PBS) buffer and centrifuged at 1300 rpm for 3 min. Then, 200 μL of 1× binding buffer and 10 µL of Annexin V-APC were added and mixed for 15 min at room temperature in the dark. The results were determined on a flow cytometer (BD, San Jose, CA, USA).

### 2.10. Cell Migration

A Transwell kit (8-μm pores, Corning Inc., Corning, New York, NY, USA) was used for cell migration analysis. A total of 100 µL of serum-free IMDM was added to the upper chamber and placed in the incubator at 37 °C for 1 h. The medium in the upper chamber was then removed and 1 × 10^5^ cells with serum-free medium were added to the coated filter membrane, and 600 µL of IMDM with 30% FBS was added to the lower chamber. The cells were incubated for another 18 h. Then, the chamber was placed in 4% paraformaldehyde solution for 30 min and the migrated cells were stained with crystal violet. Nine random regions were selected to count the cell number.

### 2.11. Colony Formation Assay

GIST-T1 cells were inoculated in 6-well plates at a density of 1000 cells/well and cultured continuously for 14 days, with medium changes every four days. Then, the cells were washed twice with PBS and 4% paraformaldehyde was added to each hole at 4 °C for 1 h. Next, the cells were stained with 0.1% crystal violet staining solution for 10–20 min. Finally, the number of cell colonies containing > 50 cells in each well was calculated and photographed.

### 2.12. Real-Time Quantitative Polymerase Chain Reaction (qPCR)

Total RNA was isolated from GIST-T1 using Trizol reagent (SuperfecTRI, Shanghai, China) following the manufacturer’s instructions. Complementary DNA (cDNA) was synthesized from 1 μg of total RNA using the PrimeScript™ RT Reagent Kit (Takara, Kusatsu, Japan) and following the manufacturer’s protocol, with reverse transcription performed at 37 °C for 15 min and 85 °C for 5 s. Gene-specific primers were used for amplification: CTD-2245E15.3-F: GTCACATCGGTCAAACCCTAC; CTD-2245E15.3-R: GTTCCGTTCTGGAGTTAGTCG; β-actin-F: GCGTGACATTAAGGAGAAGC; and β-actin-R: CCACGTCACACTTCATGATGG. Real-time qPCR was performed using SYBR Green qPCR MasterMix (Bio-Rad, Hercules, CA, USA) on a CFX96 Real-Time PCR Detection System (Bio-Rad) with the following cycling conditions: initial denaturation at 95 °C for 3 min, followed by 40 cycles of 95 °C for 10 s, 60 °C for 30 s, and 72 °C for 30 s. The CT values were normalized to the value of β-actin, and the relative RNA expression levels of the genes were calculated using the 2^−∆∆Ct^ method.

### 2.13. RNA In Situ Hybridization

RNA in situ hybridization was performed on formalin-fixed, paraffin-embedded (FFPE) tissue sections (4 μm) using the RNAscope^®^ 2.5 HD Detection Kit (Advanced Cell Diagnostics, Newark, CA, USA) according to the manufacturer’s instructions. After deparaffinization, target retrieval and protease treatment, sections were hybridized with a specific probe targeting CTD-2245E15.3 at 40 °C for 2 h. Signal amplification and chromogenic detection were subsequently performed. Positive (PPIB) and negative (DapB) control probes were included. Slides were counterstained with hematoxylin and examined under a light microscope. The shRNA target sequence and RNAscope probe region were designed based on the annotated CTD-2245E15.3 transcript in the Ensembl database. RNA in situ hybridization signals were evaluated by experienced gastrointestinal pathologists who were blinded to clinical outcomes, with stromal and inflammatory areas excluded from scoring.

### 2.14. Statistical Analysis

Continuous variables are expressed as mean ± standard deviation (SD). The statistical differences between the two groups were analyzed using the two-tailed Mann–Whitney U test, and comparisons among multiple groups were performed using the Kruskal–Wallis test. With *p* < 0.05 considered statistically significant. All statistical analyses were performed using GraphPad Prism 8.0.2 (GraphPad Software, San Diego, CA, USA). Kaplan–Meier method with log-rank test was utilized for survival analysis. Univariate and multivariate Cox proportional hazards regression analyses were performed to identify independent prognostic factors. Two-sided *p* < 0.05 was considered statistically significant. Statistical analysis was performed using SPSS 26.0 (IBM Corp., Armonk, NY, USA) and R 4.3.0 (http://www.r-project.org/) software.

## 3. Results

### 3.1. Screening Hub lncRNAs

**Figure 2:**
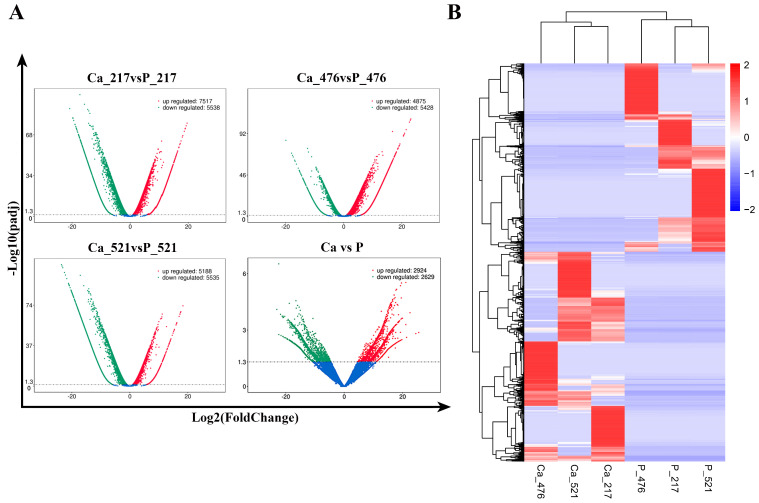
(**A**): Expression profiles of lncRNAs in gastrointestinal stromal tumor samples and adjacent normal tissues. Volcano plot showing differential lncRNA expression between adjacent normal tissues and GIST tissues. The x-axis represents the log2 fold change (log2FC), and the y-axis represents the −log10 of the adjusted *p* value (Padj). Differentially expressed lncRNAs were defined as those with |log2FC| ≥ 1 and Padj < 0.05. Green dots represent significantly down-regulated genes, red dots represent significantly up-regulated genes, and blue dots represent those that did not change significantly. (**B**): Cluster analysis of differentially expressed lncRNAs. Horizontal coordinates indicate sample names, and vertical coordinates indicate differential genes. On the left side, genes are clustered based on the degree of similarity in expression, and on the top side, cluster samples based on the similarity of expression profiles. Blue to red represents a gradual up-regulation of expression. P, paratumor normal tissue samples; Ca, gastrointestinal stromal tumor samples; lncRNA, long non-coding RNA.

The proportions of the different types of lncRNAs obtained are shown in Figure 2. Volcano plots containing differentially expressed lncRNAs were generated based on P and fold change (FC) values and used to illustrate lncRNAs that were differentially expressed between the three datasets (Figure 2A). The threshold set for up- and down-regulated genes was a FC ≥ 2.0 and a Padj ≤ 0.05. A total of 13,055 differentially expressed LncRNAs were found between the CA-217 and P-217 groups, among which 7517 were up-regulated, and 5538 were down-regulated. A total of 4875 up- and 5428 down-regulated LncRNAs were found between the CA-476 and P-476 groups, and 10,723 differentially expressed LncRNAs were found between the CA-521 and P-521 groups, of which 5188 were up-, and 5535 were down-regulated. For the original sequencing data, the three samples of tumor and adjacent normal tissues were combined and analyzed, and the results showed that 2924 were up- and 2629 were down-regulated (Figure 2A). Hierarchical clustering was used to reveal distinguishable gene expression patterns between samples (Figure 2B), with a common set of up- and down-regulated genes found in the three sets of differential genes. Given that lncRNAs are engaged in various physiological activities, especially in cell differentiation and proliferation, differentially expressed lncRNAs in GISTs and normal paratumor tissues may be involved in the tumorigenesis mechanism.

### 3.2. GO and KEGG Enrichment Analysis of Differentially Expressed lncRNAs

**Figure 3:**
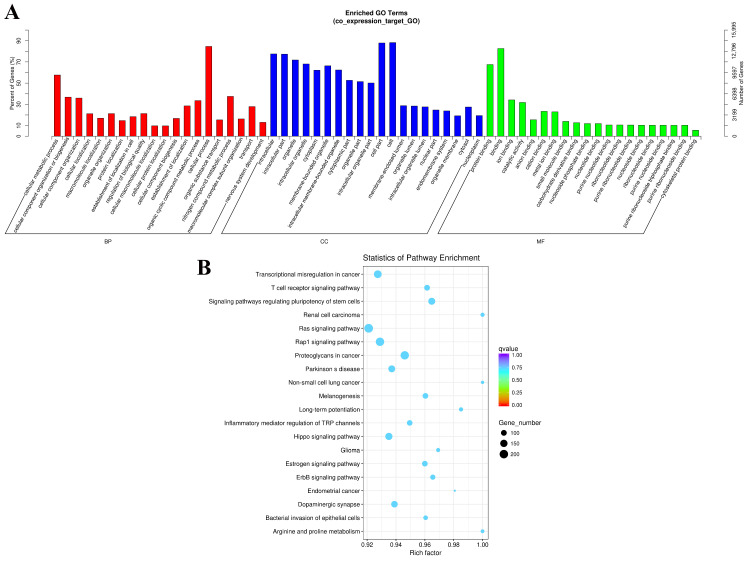
GO enrichment and KEGG pathway analysis of differentially expressed lncRNAs between gastrointestinal stromal tumor samples and adjacent normal tissues. (**A**): GO enrichment analysis; The abscissa represents the enrichment factor, while the ordinate represents a GO term. (**B**): KEGG pathway analysis. The abscissa indicates the gene numbers, while the ordinate indicates the detailed terms. GO, Gene Ontology; KEGG, Kyoto Encyclopedia of Genes and Genomes; lncRNA, long non-coding RNA.

As shown in Figure 3A, in the three categories of genes, molecular functions (MFs), biological processes (BPs), and cellular components (CCs), differentially expressed genes are mainly enriched in cellular processes, protein binding, cellular metabolic processes, and nitrogen compound metabolic processes.

KEGG enrichment analysis showed that these differentially expressed genes were significantly enriched in many pathways, including transcriptional misregulation in cancer; Arginine and proline metabolism; the ErbB, Hippo, Rap1, and Ras signaling pathways, regulating stem cell pluripotency; and the T cell receptor signaling pathway (Figure 3B).

### 3.3. Target lncRNA Screening

**Figure 4:**
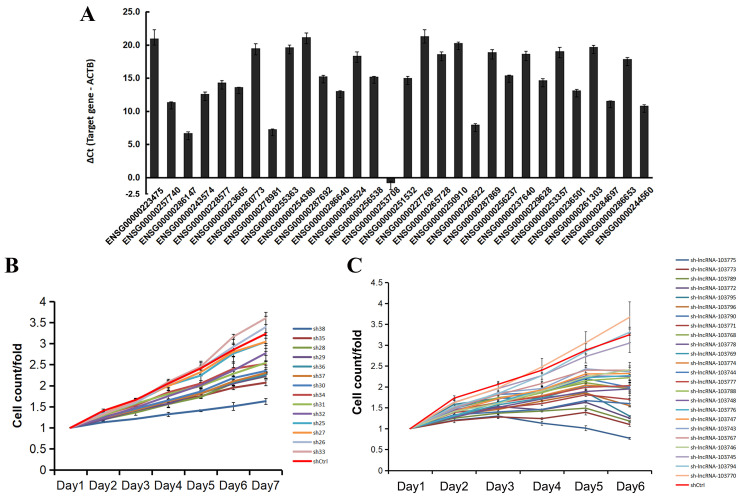
Selection of target genes by high-content screening (HCS). (**A**): The expression of the top 30 differentially expressed lncRNAs in GIST-T1. (**B**): The cell proliferation curves of the 14 differentially expressed lncRNAs. (**C**): The cell proliferation curves of the specific target of the top four anti-proliferation positive lncRNAs (sh28, sh29, sh35, sh38) and sh21, sh22, sh23, and sh24 (three RNA interference targets for each gene).

First, we selected the top differentially expressed lncRNAs and performed expression detection in GIST-T1 cells (Figure 4A), then secondly performed HCS on those lncRNAs highly expressed in GIST-T1 cells to determine the effect of gene knockdown on cell proliferation, and to preliminarily screen out the genes with an obvious proliferation promotion phenotype.

Since the sh21, sh22, sh23, and sh24 infection efficiencies were less than 10% after infecting the GIST-T1 cells, single-target HCS was subsequently performed on these four genes. HCS results showed that among the other 14 experimental groups to be tested, the experimental groups with a fold change (control/experimental group) in proliferation ≥ 1.45 were sh38, sh35, sh28, and sh29 (Table 2). The cell proliferation curves are shown in Figure 4B. Then, single-target HCS was performed on the first four anti-proliferation positive lncRNAs (sh28, sh29, sh35, sh38) and sh21, sh22, sh23, and sh24, and the results are shown in Table 3 and Figure 4C. Thereafter, qPCR verification was performed on single targets with FC values > 1.4. We chose CTD-2245E15.3 (ENST00000503113.1/sh23/sh-lncRNA-103772) with a knockdown efficiency > 50% for further functional verification.

### 3.4. CTD-2245E15.3 Knockdown Suppresses GIST Cell Proliferation

**Figure 5:**
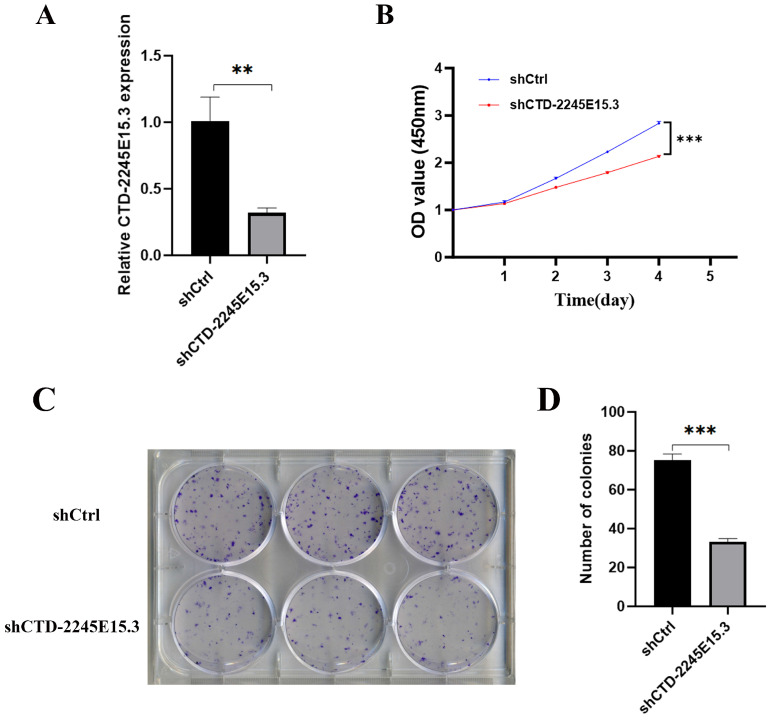
CTD-2245E15.3 knockdown inhibits gastrointestinal stromal tumor cell proliferation. (**A**): The efficiency of shRNA-mediated CTD-2245E15.3 knockdown was verified by real-time quantitative polymerase chain reaction. (**B**): CCK-8 was used for cell proliferation analysis. (**C**,**D**): CTD-2245E15.3 gene knockdown can reduce the GIST-T1 cell colony number. ***, *p* < 0.001; **, *p* < 0.01.

To verify the effect of CTD-2245E15.3 on tumor cell proliferation, GIST-T1 cells were infected with lentivirus-mediated shRNA targeting CTD-2245E15.3 in the experimental group and negative-control lentivirus in the control group. Firstly, the efficiency of knockdown was verified by qPCR. In GIST-T1 cells stably infected with Lenti-shCTD-2245E15.3, CTD-2245E15.3 was significantly down-regulated compared with the control group (Figure 5A). The CCK-8 assay showed that GIST-T1 cell proliferation rate was significantly inhibited after CTD-2245E15.3 knockdown (Figure 5B). Moreover, after 3 days of shRNA lentivirus infection, the cells were spread on 6-well plates, and after 14 more days, the GIST cell colony number in the shCTD-2245E15.3 group was significantly less than the control group (Figure 5C). These results indicate that CTD-2245E15.3 promotes GIST-T1 cell proliferation rate. The results in GIST-882 cells (Figure S1) are consistent with those observed in GIST-T1.

### 3.5. CTD-2245E15.3 Knockdown Reduces Invasive Ability and Induces Apoptosis of GIST Cells

**Figure 6:**
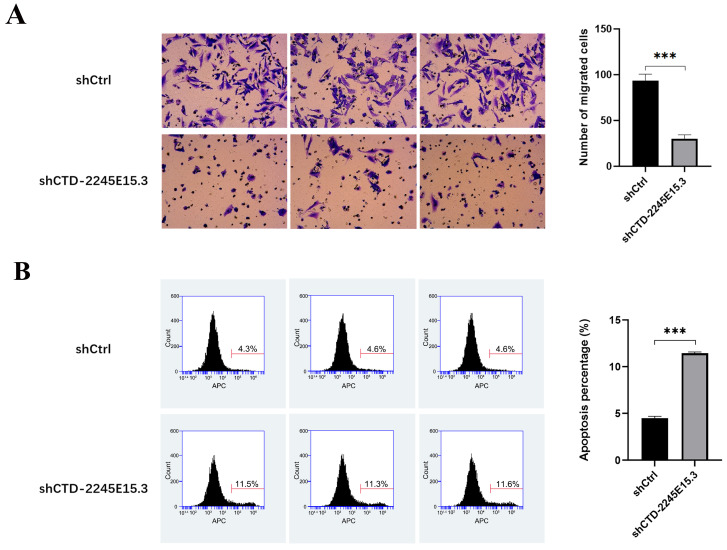
CTD-2245E15.3 gene knockdown inhibits the malignant behaviors of gastrointestinal stromal tumor cells. (**A**): Transwell test for migration detection; (**B**): The apoptosis rate was detected by flow cytometry. ***, *p* < 0.001.

The results in Figure 6A demonstrated that the migratory ability in the shCTD-2245E15.3 group was significantly lower than that of the control group, suggesting that CTD-2245E15.3 is related to the GIST-T1 cell metastatic capacity. To evaluate the effect of lncRNA expression on apoptosis, the degree of apoptosis after CTD-2245E15.3 gene knockdown was analyzed by flow cytometry. Annexin V-APC staining found that the percentage of apoptotic cells increased significantly in the shCTD-2245E15.3 group compared with the shCtrl group (Figure 6B), indicating that CTD-2245E15.3 may prevent GIST-T1 cell apoptosis. The same results were observed in GIST-882 cells (Figure S2).

### 3.6. CTD-2245E15.3 Expression Was Associated with Adverse Clinical Characteristics and Worse Prognosis

**Figure 7:**
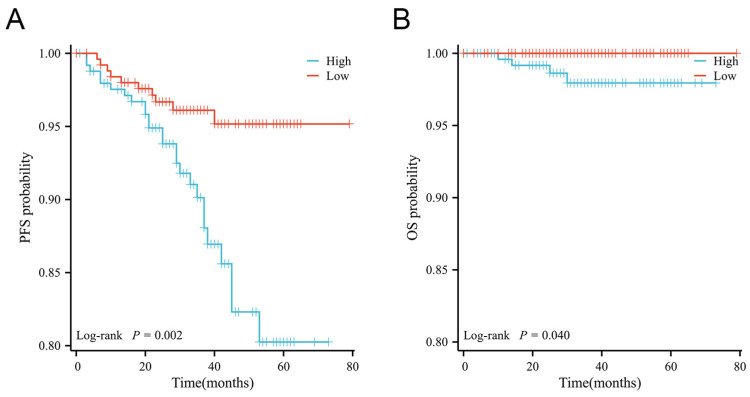
Kaplan–Meier survival analysis of progression-free survival (**A**) and overall survival (**B**) stratified by CTD-2245E15.3 expression in patients with gastrointestinal stromal tumors.

The association between CTD-2245E15.3 expression and clinicopathological features was analyzed to reveal the clinical relevance. Higher CTD-2245E15.3 expression was associated with adverse clinical characteristics, including larger tumor size and higher mitotic index (Table 4). Kaplan-Meier analysis showed that patients with high CTD-2245E15.3 expression had shorter PFS and OS (Figure 7). Furthermore, multivariate Cox proportional hazards analysis, adjusting for tumor size, mitotic index, and tumor morphology, confirmed that high CTD-2245E15.3 expression was an independent predictor of shorter PFS (HR = 2.519, 95% CI: 1.208–5.255, *p* = 0.014) (Table S1). These results support the potential prognostic relevance of CTD-2245E15.3 in GISTs.

## 4. Discussion

GIST, the most common tumor of mesenchymal origin in the gastrointestinal tract, can be graded for risk based on tumor location, size, mitotic count, and the presence or absence of rupture. Considering both practicality and convenience, the 2008 modified NIH classification is most commonly employed [15]. Given that intermediate- and high-risk GISTs are more likely to develop implantation and metastasis, we conducted lncRNA sequencing on frozen specimens from three pairs of intermediate- and high-risk tumors along with matched peritumoral tissues. The specimens originated from the stomach, duodenum, and small intestine, respectively. While the inclusion of tissues from different anatomical sites introduces biological variability, it also serves as a strength of this study. Identification of dysregulated lncRNAs across these anatomically distinct samples suggests that these genes, including CTD-2245E15.3, may represent fundamental oncogenic drivers common to high-risk GISTs regardless of their site of origin. This implies that CTD-2245E15.3 could potentially serve as a broad-spectrum therapeutic target, although future studies with larger, site-stratified cohorts are needed to dissect site-specific nuances.

There are several previous reports on lncRNA sequencing and differential screening of GISTs and normal paratumor tissue. Jingyi Yan et al. compared lncRNA expression in primary GISTs, secondary imatinib-resistant GISTs, and normal gastric wall tissues, and performed bioinformatic analysis of differentially expressed lncRNAs to predict their potential roles in tumor development, recurrence, metastasis, and drug resistance, but did not perform functional validation and mechanistic exploration [16]. Ugne Gyvyte et al. examined GIST and peritumor tissue and identified nine significantly dysregulated lincRNAs, of which MALAT1, H19, and FENDRR were validated by quantitative real-time PCR, and their correlation with GIST-related oncogenes and miRNAs was analyzed. However, the study used formalin-fixed paraffin specimens with severe RNA degradation, which may have affected the reliability of the results [17]. Xiaonan Yin et al. performed RNA sequencing of GISTs with different risk grades to demonstrate the lncRNA expression profile during malignant transformation of GISTs. They showed that DNM3OS correlated with tumor volume, mitotic count, risk classification, and survival in GIST patients, and that it promoted GIST cells’ proliferation and mitosis by regulating the expression of GLUT4 and CD36 [18]. Yebo Shao et al. [19] performed high-throughput RNA sequencing of ten pairs of GIST tissues and adjacent normal tissues to characterize the transcription and dysregulation of lncRNAs in GISTs. The research identified RP11-616M22.7 as being associated with imatinib resistance, a lncRNA that binds to the RASSF1 protein and is involved in regulating the Hippo signaling pathway. Given the relatively limited availability of lncRNA data for GISTs in commonly used databases such as TCGA, Oncomine, and GEO, using freshly frozen surgical specimens to obtain first-hand sequencing data for a particular patient population can help to gain insight into the pathogenesis and therapeutic targets of GISTs.

The sequencing results of our study demonstrated many differentially expressed lncRNAs, and the GO and KEGG enrichment analyses provided preliminary insights into the biochemical, metabolic, and signaling pathways that may be involved in these differentially expressed lncRNAs. GO and KEGG analyses revealed that differentially expressed lncRNAs were mainly enriched in cellular metabolism-related pathways, and metabolic reprogramming was closely associated with tumor occurrence and development [20].

However, it is worth noting that the degree of difference in lncRNA expression does not necessarily correlate with its importance in tumor development, and the bioinformatics analysis results are not sufficient to clarify the physiopathological functions and acting mechanisms of lncRNAs [21]. We combined the degree of expression variation and HCS to initially select the lncRNA CTD-2245E15.3, and performed CCK-8 cell proliferation, transwell cell migration assay, colony formation assay, and cell apoptosis analysis to verify that knockdown of this lncRNA inhibits the proliferation and migration ability of GIST-T1 cells and induces apoptosis. The results in GIST-882 cells are consistent with those observed in GIST-T1, showing that CTD-2245E15.3 knockdown suppresses proliferation and migration and promotes apoptosis. These results suggest that CTD-2245E15.3 may be associated with the biological behaviors of GISTs, such as tumorigenesis, invasion, and metastasis.

Reports on the lncRNA CTD-2245E15.3 are relatively rare. Chen Wang et al. employed lncRNA microarray analysis to analyze differentially expressed lncRNAs in non-small-cell lung cancer (NSCLC), from which CTD-2245E15.3, with the highest expression level, was selected and its tumor growth-promoting effect was demonstrated in in vitro and in vivo experiments [22]. The authors also explored the mechanism of CTD-2245E15.3 and found that the down-regulated genes after the lncRNA knockdown were mainly involved in biomolecular metabolic processes and fatty acid synthesis through GO analysis. The study confirmed that CTD-2245E15.3 binds to two key enzymes of anabolic metabolism, acetyl-CoA carboxylase 1 (ACC1) and pyruvate carboxylase (PC), and enhances cell proliferation by promoting anabolism, thus exerting pro-tumorigenic effects.

Although bulk RNA sequencing enables comprehensive transcriptomic profiling, it inherently reflects composite signals derived from tumor cells as well as surrounding stromal and immune components. Therefore, differential expression identified from bulk tissue may partially capture microenvironmental influences rather than tumor cell–intrinsic alterations alone. To mitigate this limitation, we performed RNA in situ hybridization in an independent clinical cohort, which demonstrated that CTD-2245E15.3 expression was predominantly localized within tumor regions. Nevertheless, we acknowledge that bulk transcriptomic analyses cannot fully disentangle cellular heterogeneity. Future studies integrating single-cell RNA sequencing or computational deconvolution approaches would further clarify the cell-type–specific expression pattern and functional contribution of CTD-2245E15.3 in GIST progression.

Our study depicted a panel of differentially expressed lncRNAs from different sites of intermediate- to high-risk GIST, which were mostly enriched in metabolism-related pathways as shown by GO and KEGG analyses. We selected CTD-2245E15.3 based on the degree of differential expression and HCS results, in which it was shown to promote GIST cell proliferation and migration and reduce apoptosis by in vitro functional cellular assays. To further strengthen the translational relevance of CTD-2245E15.3, future mechanistic studies are planned to elucidate its molecular interactions and regulatory networks in GISTs. Specifically, we aim to perform RNA pull-down and RNA immunoprecipitation (RIP) assays to identify binding proteins and potential effector molecules. Additionally, overexpression and complementary knockdown experiments will be conducted to validate the causal roles of CTD-2245E15.3 in modulating proliferation, migration, and apoptosis. These planned studies will provide mechanistic insights and may inform the development of targeted therapeutic strategies in the future. The main challenge in lncRNA research is determining how to select effective therapeutic targets or tumor markers from the vast amount of sequencing data. We will continue to attempt to validate new lncRNAs with therapeutic or diagnostic potential among differentially expressed lncRNAs, and will validate the expression of lncRNAs in more clinical cases and their relationship with survival, recurrence, metastasis, and drug resistance.

## 5. Limitations

This study has several limitations. First, transcriptomic profiling was performed on bulk tumor tissues, which may reflect composite cellular signals and be influenced by tumor microenvironment heterogeneity. Although RNA in situ hybridization provided spatial validation, single-cell transcriptomics or computational deconvolution would allow more precise resolution of tumor versus stromal/immune contributions. Second, functional assays were conducted in two GIST cell lines using shRNA-mediated knockdown, and mechanistic conclusions remain primarily based on in vitro phenotypes; additional isoform-specific validation, complementary knockdown or overexpression strategies, and in vivo studies are warranted to fully substantiate causality. Third, prognostic analyses were exploratory, and larger, prospective cohorts will be necessary to confirm the independent clinical relevance of CTD-2245E15.3.

## 6. Conclusions

In summary, this study identifies long non-coding RNA CTD-2245E15.3 as a novel functional regulator in GISTs. CTD-2245E15.3 was found to be upregulated in intermediate- to high-risk GISTs and to promote tumor cell proliferation and migration while inhibiting apoptosis in vitro. These findings suggest that CTD-2245E15.3 may play an oncogenic role in GIST progression and could serve as a potential therapeutic target or biomarker. Further studies are warranted to elucidate the underlying molecular mechanisms and to explore their clinical and translational relevance.

## Figures and Tables

**Table 1:** The detailed clinicopathological characteristics of the three pairs of sequencing samples.

Patients	Sex	Age (Years)	Location	Tumor Size (cm)	Morphology	Mitotic Index/50 HPF	NIH Risk Grade	Gene Mutation
217	Female	53	Duodenum	7.5 × 6.5	Spindle	0	Moderate	C-kit exon 11
476	Female	38	Stomach	6.5 × 5	Spindle	29	High	C-kit exon 11
521	Male	67	Small intestine	5.5 × 5	Spindle	3	High	C-kit exon 11

**Table 2:** HCS results of the 18 top differentially expressed lncRNAs.

Gene	Virus Number	shRNA Number	Fold Change (Control/Experimental)
Ctrl	Non-targeting shRNA	shCtrl	1
ENST00000546339.2	PSC103743mix	sh21	-
ENST00000534086.1	PSC103746mix	sh22	-
ENST00000503113.1	PSC103770mix	sh23	-
ENST00000662236.1	PSC103776mix	sh24	-
ENST00000660031.1	psc103779mix	sh25	1.06
ENST00000415249.1	psc103782mix	sh26	0.95
ENST00000415275.1	psc103785mix	sh27	1.06
ENST00000467017.2	psc103788mix	sh28	1.45
ENST00000416232.5	psc103794mix	sh29	1.45
ENST00000523733.1	psc103749mix	sh30	1.38
ENST00000625026.1	psc103755mix	sh31	1.27
ENST00000650850.1	psc103758mix	sh32	1.16
ENST00000658626.1	psc103761mix	sh33	0.9
ENST00000436056.1	psc103764mix	sh34	1.28
ENST00000536492.1	psc103767mix	sh35	1.56
ENST00000664749.1	psc103752mix	sh36	1.43
ENST00000546789.1	psc103791mix	sh37	1.4
ENST00000537655.2	psc103773mix	sh38	1.98

**Table 3:** Single-target HCS of the first four anti-proliferation positive lncRNAs (sh28, sh29, sh35, sh38) and sh21, sh22, sh23, and sh24.

Gene	Number	Sequence	Fold Change (Control/Experimental)
	sh-lncRNA-103743	GCCAACAGAATCAGAGCAAAT	1.37
sh21	sh-lncRNA-103744	GGAGCTGTTGCCCAAAGAATT	1.63
(ENST00000546339.2)	sh-lncRNA-103745	GCTCACCTGCTGGTGATAAAC	1.06
	sh-lncRNA-103746	GCAGCCAAGTTTGGCTACAGC	1.35
sh22	sh-lncRNA-103747	GCTCCCTAGTCTGGACTCGCT	1.4
(ENST00000534086.1)	sh-lncRNA-103748	GCTCATCACTGGGAAAGATGG	1.44
	sh-lncRNA-103770	GCCAGCTAACGTGTTCTAATA	0.88
sh23	sh-lncRNA-103771	GGTCCTACAGACAATGAATAT	1.91
(ENST00000503113.1)	sh-lncRNA-103772	GCATTCTTAGGAAATGCCTCC	2.6
	sh-lncRNA-103776	GGTCTGGTCTAATCCACTTTA	1.43
sh24	sh-lncRNA-103777	GCTCAGCAAAGTGATGATATA	1.6
(ENST00000662236.1)	sh-lncRNA-103778	GCCCAATTCTTCTCCACAACA	1.66
	sh-lncRNA-103788	GCACAGATCAGATGCCCATTC	1.46
Sh28	sh-lncRNA-103789	GCAATCGTCAGAGAAGAATGG	2.76
(ENST00000467017.2)	sh-lncRNA-103790	GCACCTTGAAGATTTGCAAGA	2.02
	sh-lncRNA-103794	GGAGTTCTGCAAATGTGTTGT	0.98
sh29	sh-lncRNA-103795	GGAAGCATGTGGAGATCATTT	2.51
(ENST00000416232.5)	sh-lncRNA-103796	GGTAGAAGGTATCTGTCATTC	2.09
	sh-lncRNA-103767	GCACTGTGGTGAGGATGCAAA	1.35
sh35	sh-lncRNA-103768	GGAACCAATCTAGCTGACACT	1.67
(ENST00000536492.1)	sh-lncRNA-103769	GGACTTCAAACCTCCAGTATT	1.64
	sh-lncRNA-103773	GGAGGAGAGATGCAGTCTTAG	2.94
sh38	sh-lncRNA-103774	GCAGGAATAAAGAAGCCTATA	1.63
(ENST00000537655.2)	sh-lncRNA-103775	GCTGATTCTTAAAGGGCTAGA	4.24

**Table 4:** Association between CTD-2245E15.3 expression and clinicopathological characteristics in patients with gastrointestinal stromal tumors (GISTs).

Factors	CTD-2245E15.3		*p*-Value
	LOW (n = 254)	HIGH (n = 253)	
Sex			**0.** **002**
male	130 (51.2%)	164 (64.8%)	
female	124 (48.8%)	89 (35.2%)	
Age(years)			0.506
≤60	137 (53.9%)	129 (51.0%)	
>60	117 (46.1%)	124 (49.0%)	
Location			0.363
Stomach	164 (64.6%)	173 (68.4%)	
Non-stomach	90 (35.4%)	80 (31.6%)	
Tumor size			**0.022**
≤5 cm	163 (64.2%)	137 (54.2%)	
>5 cm	91 (35.8%)	116 (45.8%)	
Mitotic index			**0.006**
≤2/50 HPF	193 (76.0%)	164 (64.8%)	
>2/50 HPF	61 (24.0%)	89 (35.2%)	
Morphology			0.469
Spindle	213 (83.9%)	206 (81.4%)	
Epithelioid and Mixed	41 (16.1%)	47 (18.6%)	

Bold values indicate statistical significance (*p* < 0.05).

## Data Availability

The raw data supporting the conclusions of this article will be made available by the corresponding authors on request.

## Supplementary Materials

The following supporting information can be downloaded at: https://www.mdpi.com/article/10.3390/biomedicines14030514/s1, Table S1: Univariate and multivariate Cox regression assessing associations between parameters and PFS; Figure S1: CTD-2245E15.3 knockdown inhibits the proliferation of GIST-882 cells; Figure S2: CTD-2245E15.3 gene knockdown inhibits the malignant behaviors of GIST-882 cells.
