# Supplementary material for: Long Non-Coding RNA CTD-2245E15.3 Drives Proliferation and Migration in Gastrointestinal Stromal Tumors
Source: Biomedicines. 2026 Feb 26;14(3):514. doi: 10.3390/biomedicines14030514 (PMC13023645; doi:10.3390/biomedicines14030514)
Supplement: Supplementary file 1 [file biomedicines-14-00514-s001.zip › biomedicines-4124923-supplementary.pdf]

Long Non-coding RNA CTD-2245E15.3 Drives Proliferation and Migration in Gastrointestinal Stromal Tumors

Supplementary Tables and Figures

Table S1. Univariate and multivariate Cox regression assessing associations between parameters and PFS.

Variable	Univariate analysis		Multivariate analysis	
	HR (95%CI)	P-value	HR (95%CI)	P-value
Sex		0.756		
female	1			
male	1.112（0.569-2.174）			
Age(years)		0.124		
≤60	1			
>60	1.693（0.866-3.309）			
Location		0.800		
Stomach	1			
Non-stomach	1.091（0.557-2.135）			
Tumor size		**0.030**		0.538
≤5cm	1		1	
>5cm	2.079（1.072-4.034）		1.244（0.621-2.491）	
Mitotic index		**<0.001**		**<0.001**
≤2/50HPF	1		1	
>2/50HPF	4.991（2.496-9.982）		3.943（1.897-8.196）	
Morphology		**0.005**		**0.048**
Spindle	1		1	
Epithelioid and Mixed	2.638（1.335-5.212）		2.005（1.007-3.993）	
CTD - 2245E15.3		**0.003**		**0.014**
Low	1		1	
High	2.976（1.433-6.178）		2.519（1.208-5.255）	

Bold values indicate statistical significance (P < 0.05).

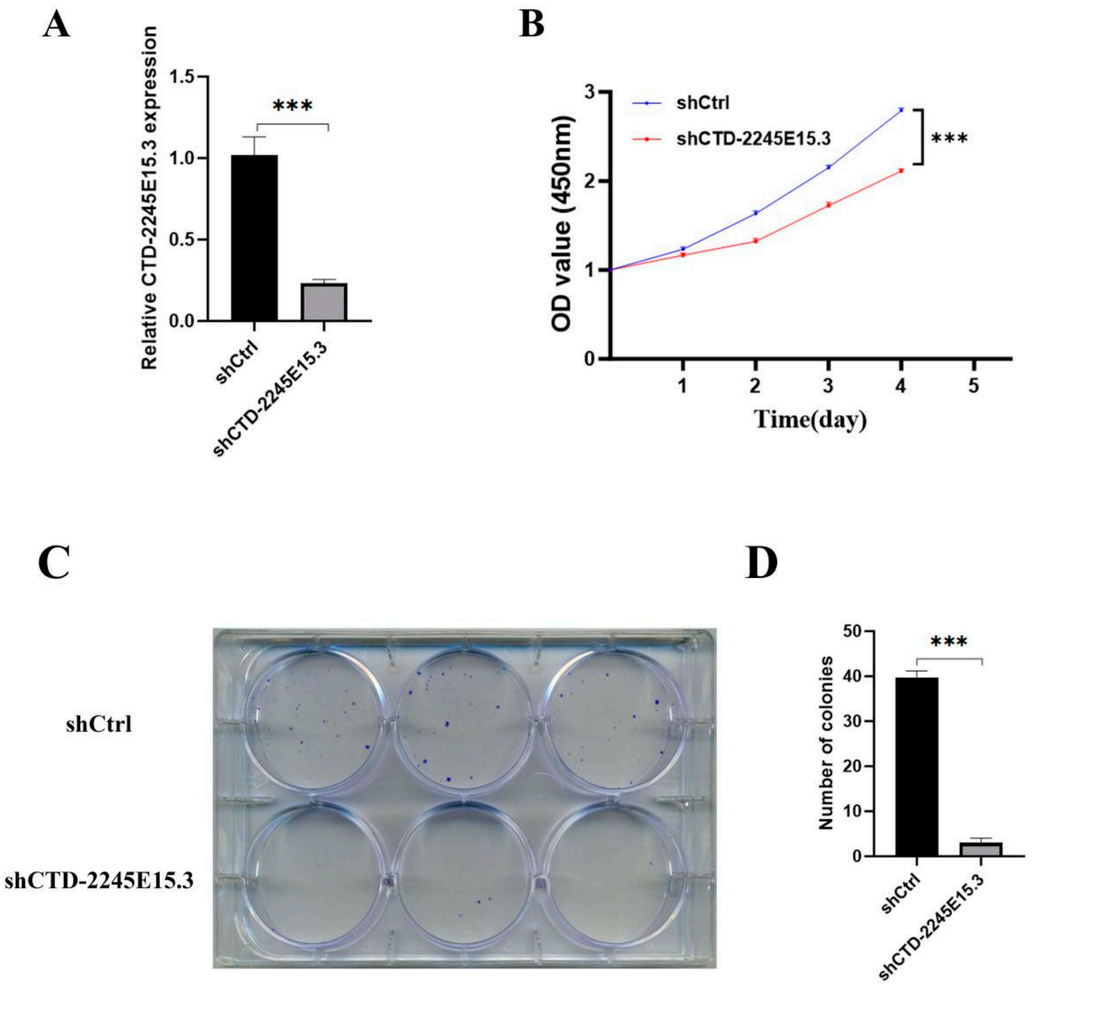

Figure S1. CTD-2245E15.3 knockdown inhibits the proliferation of GIST-882 cells. A: The efficiency of shRNA-mediated CTD-2245E15.3 knockdown was verified by real-time quantitative polymerase chain reaction. B: CCK-8 was used for cell proliferation analysis. C-D: CTD-2245E15.3 gene knockdown can reduce the colony number of GIST-882 cells. ***, P<0.001.

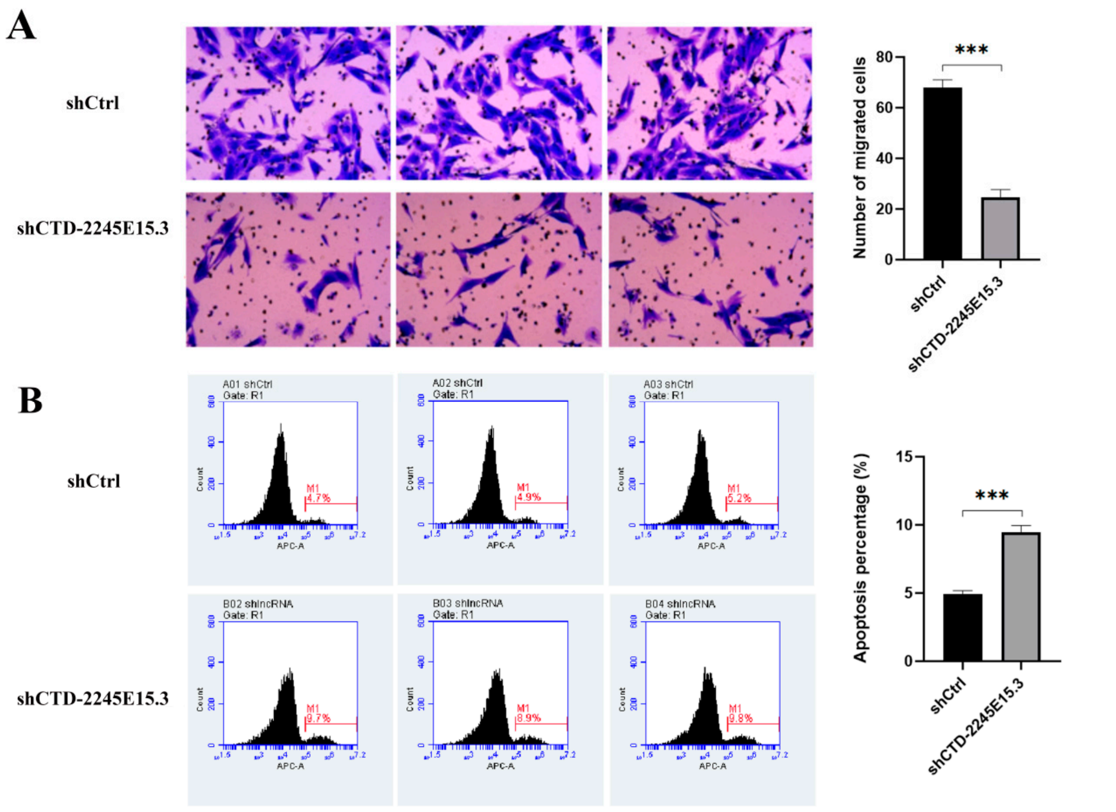

Figure S2. CTD-2245E15.3 gene knockdown inhibits the malignant behaviors of GIST-882 cells. A Transwell test for migration detection; B The apoptosis rate was detected by flow cytometry. ***, P<0.001.
